# Buildings Contemplated

**Published:** 1914-04-18

**Authors:** 


### Buildings Contemplated.

Albury.—A gift of £1,000 has been made by Mr. James Mitchell, of Table Top, for building a new hospital at Albury, New South Wales.

Bexhill.—The Town Council have decided to apply to the Local Government Board for sanction to borrow £6,420 for a new isolation hospital.

Bradwell.—The Bradwell Joint Hospital Committee have received sanction from the Local Government Board to borrow £7,448 for alterations and extensions to the hospital.

Burton-on-Trent.—The General Committee of the Burton-on-Trent Infirmary have accepted tenders, amounting to £7,490, for the extension scheme.

Cardiff.—The Board of Guardians invite tenders for extensions to the nurses' room at the Hospital Headquarters Homes, Ely, near Cardiff. Specifications may be obtained from Mr. Arthur J. Harris, clerk, Union Offices, Queen's Chambers, Cardiff.

Hemel Hempstead.—The Joint Isolation Hospital Board have passed a resolution authorising the borrowing of £5,862 from the Public Works Loan Commissioners for a new hospital.

Heston.—The Middlesex County Council propose to purchase for £33,000 the Rectory Farm and part of Vicarage Farm, Heston, as a site for a new county asylum.

Kettering.—Plans have been prepared for the extension of the infectious diseases hospital. The estimated cost is £1,252.

Nantyglo.—Tenders are invited for extensions, alterations, and additions to the Blaina and District Cottage Hospital, near Nantyglo. Mr. E. A. Johnson, F.R.I.B.A., Abergavenny, is the architect, from whom quantities may be obtained.

Saltburn.—Plans have been approved for an isolation hospital for the Urban District Council.

Torquay.—The Devon County Council Public Health Committee propose that a main dispensary and tuberculosis hospital be provided at Torquay; proposals have been submitted to the Local Government Board for acquiring premises at a cost of £1,600.

